# Transsulfuration is an active pathway for cysteine biosynthesis in *Trypanosoma rangeli*

**DOI:** 10.1186/1756-3305-7-197

**Published:** 2014-04-24

**Authors:** Ibeth Romero, Jair Téllez, Lais Eiko Yamanaka, Mario Steindel, Alvaro José Romanha, Edmundo Carlos Grisard

**Affiliations:** 1 Laboratórios de Protozoologia e de Bioinformática, Departamento de Microbiologia, Imunologia e Parasitologia, Centro de Ciências Biológicas, Universidade Federal de Santa Catarina, Florianópolis, SC 88040-970, Brasil; 2 Centro de Pesquisas René Rachou, Fiocruz, Belo Horizonte, MG, Brasil

**Keywords:** Cysteine biosynthesis, Cystathionine β-synthase, Cysteine synthase, *T. rangeli*, Thiol metabolism, Antioxidant defence

## Abstract

**Background:**

Cysteine, a sulfur-containing amino acid, plays an important role in a variety of cellular functions such as protein biosynthesis, methylation, and polyamine and glutathione syntheses. In trypanosomatids, glutathione is conjugated with spermidine to form the specific antioxidant thiol trypanothione (T[SH]_2_) that plays a central role in maintaining intracellular redox homeostasis and providing defence against oxidative stress.

**Methods:**

We cloned and characterised genes coding for a cystathionine β-synthase (CβS) and cysteine synthase (CS), key enzymes of the transsulfuration and assimilatory pathways, respectively, from the hemoflagellate protozoan parasite *Trypanosoma rangeli*.

**Results:**

Our results show that *T. rangeli* CβS (TrCβS), similar to its homologs in *T. cruzi*, contains the catalytic domain essential for enzymatic activity. Unlike the enzymes in bacteria, plants, and other parasites, *T. rangeli* CS lacks two of the four lysine residues (Lys^26^ and Lys^184^) required for activity. Enzymatic studies using *T. rangeli* extracts confirmed the absence of CS activity but confirmed the expression of an active CβS. Moreover, CβS biochemical assays revealed that the *T. rangeli* CβS enzyme also has serine sulfhydrylase activity.

**Conclusion:**

These findings demonstrate that the RTS pathway is active in *T. rangeli*, suggesting that this may be the only pathway for cysteine biosynthesis in this parasite. In this sense, the RTS pathway appears to have an important functional role during the insect stage of the life cycle of this protozoan parasite.

## Background

L-cysteine, a sulfur-containing amino acid, is indispensable for the survival of virtually all living organisms, from bacteria to higher eukaryotes. This amino acid is implicated in several processes, including the stability, structure, regulation of catalytic activity, and post-translational modification of various proteins [1]. Due to the ability of its thiol group to undergo redox reactions, L-cysteine forms the basic building block of all thiol antioxidants, acting as a direct antioxidant and also as a precursor for the biosynthesis of glutathione, trypanothione, or ovothiol [2]. In addition, cysteine is also essential for the synthesis of biomolecules, including coenzyme A, hypotaurine, taurine, and ubiquitous iron-sulphur (Fe-S) clusters, which are involved in electron transfer, redox regulation, nitrogen fixation, and regulatory process sensing [3,4].

Two different routes for cysteine biosynthesis have been described: reverse-transsulfuration (RTS) and *de novo* or assimilatory pathways. RTS has been demonstrated in fungi and mammals and includes the complete process leading to cysteine from methionine via the intermediary formation of cystathionine [5]. These reactions are catalysed by two enzymes, CβS (cystathionine β-synthase), which synthesizes cystathionine from homocysteine and serine, and CGL (cystathionine γ-lyase), which forms cysteine from cystathionine [6]. The *de novo* pathway is also catalysed by two steps starting with serine acetyltransferase (SAT) to form O-acetylserine (OAS) from L-serine and acetyl-coenzyme A. Subsequently, OAS reacts with sulfide to produce cysteine in an alanyl-transfer reaction by cysteine synthase (CS) [7]. This *de novo* pathway for cysteine biosynthesis is found in plants, bacteria, and some protozoa, such as *Entamoeba histolytica*, *Entamoeba dispar *[8], *Leishmania major *[9], and *Leishmania donovani *[10], but is absent in mammals [11]. Both CβS and CS are PLP-dependent enzymes that are evolutionary-related and in most cases some CS activity has been demonstrated for the CβS enzymes described to date [12].

It is well established that the antioxidant defence system plays a key role in the host-parasite interaction for intracellular pathogenic trypanosomatids such as *T. cruzi* and *Leishmania* spp., promoting the protection of the parasite against macrophage-derived oxygen and nitrogen-reactive species [13,14]. Among trypanosomatids, the mammalian-infective and non-pathogenic *Trypanosoma rangeli* is of growing interest because its intracellular life stage within mammalian hosts is still unknown and its sympatric occurrence with *T. cruzi *[15].

Because *T. rangeli* is required for a response to a variety of oxidative stresses in both mammalian and invertebrate hosts, the present study characterised genes encoding key enzymes of cysteine biosynthesis, a crucial precursor of trypanothione.

## Methods

### Parasites and culture

Epimastigotes of *T. rangeli* Choachí strain and *T. cruzi* Y strain were grown at 26.5°C in liver infusion tryptose medium (LIT) supplemented with 10% heat-inactivated fetal bovine serum (FBS), 100 units/mL penicillin, and 100 μg/mL streptomycin by weekly passaging [16]. Parasites were harvested at the late log phase for DNA or protein extraction as well as for thiol profiling and *in vitro* oxidative and nitrosative stress testing. Trypomastigotes of *T. rangeli* were obtained *in vitro* under conditions previously described [17].

*T. cruzi* culture-derived trypomastigotes and amastigotes were obtained from THP-1 differentiated macrophage-like cells (ATCC) infected with Y strain metacyclic trypomastigotes [18]. Briefly, THP-1 cells (ATCC) were cultured in RPMI 1640 medium supplemented with 10% FBS at 37°C in a 5% CO_2_ atmosphere and transformed to adherent macrophages using phorbol myristate acetate (50 ng/mL) for 72 h at 37°C and 5% CO_2_ prior to experiments. THP-1 macrophage-like cells were infected with *T. cruzi* trypomastigotes for 2 h at a 3:1 parasite-cell ratio and then washed to remove the extracellular parasites. After 72 h at 37°C under 5% CO_2_, the trypomastigotes were collected from the culture supernatant, centrifuged at 600 × *g* for 30 min, and then left under the same conditions for 3 h to separate the trypomastigotes from the amastigotes and cellular debris. The supernatant containing the trypomastigotes was used for protein extraction.

### Identification of *T. rangeli* CβS and CS

Both the *T. rangeli* genome and transcriptome databases (http://www.rangeli.lncc.br) [19] were searched using the TBLASTN algorithm with the protein sequences of cystathionine β-synthase (CβS) and cysteine synthase (CS) from bacteria, yeast, plants, and parasitic protozoa as queries to identify putative *T. rangeli* proteins involved in transsulfuration and assimilatory pathways. Other coding sequences for potential enzymes comprising the two biosynthetic pathways were also searched in the genome and transcriptome databases. *T. rangeli* genomic DNA (gDNA) was isolated by the phenol–chloroform method following a standard protocol [20]. The open reading frames (ORFs) of the *CβS* and *CS* genes were amplified by PCR using gene-specific primers: CBTrXhoI (5′-**CTC GAG** ACC ATG GCT CAA ACC CAC-3′) and CBTrBamHI (5′-**GGA TCC** GCG CAC CTG CTT TTT ATC C-3′) for *CβS* and CSTrNdeI (5′- **CAT ATG** GAA GCT CTC ATC GGG G-3′) and CSTrXhoI (5′- **CTC GAG** CCA GCA CCA CGG GAA GC-3′) for *CS*. Sites for restriction enzymes (included in the primer name; bolded nucleotides) were included to allow cloning. All PCR assays were carried out using a Mastercycler® Gradient (Eppendorf, Hamburg) for 30 cycles of denaturation (94°C, 1 min), annealing (60°C, 45 sec), and extension (72°C, 1 min), followed by a final extension step (72°C) for 5 min. The PCR products were cloned into the pGEM-T-Easy vector (Promega), and the resulting constructs were verified by sequencing using a Megabace 1000® DNA Analysis System with the DYEnamic ET terminators kit (GE Healthcare) according to the manufacturer’s conditions. Both DNA strands were sequenced for each clone obtained; after analysis using the Phred/Phrap/Consed package [21], only high-quality DNA sequences (Phred ≥ 20) were compared with the public databases using the GenBank BLAST algorithm.

### Protein expression and purification

The inserts corresponding to the *CβS* and *CS* ORFs cloned into pGEM-T-Easy (Promega) were excised and subcloned into the pET14b expression vector (Novagen) pre-digested with the appropriate restriction enzymes (included in the PCR primers). The resulting plasmids containing the *CβS* and *CS* genes were named pET14-TrCβS and pET14-TrCS, respectively, and re-sequenced for confirmation as described above.

The pET14-TrCβS plasmid was used to transform *E. coli* BL21 (DE3) for recombinant protein expression. Pre-inoculum was grown overnight in LB (Luria– Bertani) broth supplemented with 100 μg/mL ampicillin at 37°C and then used to inoculate fresh LB until an O.D._600_ of 0.6 was reached. The expression of recombinant CβS (rTrCβS) was induced with 1 mM isopropyl β-D-thiogalactopyranoside (IPTG) for 2 h at 37°C. The cells were harvested and resuspended in 5 mL of buffer A [50 mM sodium phosphate, 0.3 M NaCl, pH 8.0, and 25 μM pyridoxal phosphate (PLP)] containing 5 mM imidazole and then disrupted by sonication. The soluble and insoluble fractions were recovered by centrifugation at 16,000 × *g* for 30 min at 4°C [9]. rTrCβS was purified from insoluble fractions by affinity chromatography on a Ni^2+^-nitrilotriacetic (NTA) column (Qiagen) following standard procedures. Briefly, the insoluble fraction was resuspended in a buffer containing 8 M urea, 10 mM Tris, and 100 mM NaH_2_PO_4_, pH 8.0, and incubated for 1 h at 65°C to dissolve the inclusion bodies and then centrifuged (10,000 × *g* for 30 min at 4°C). The supernatants were then applied to the Ni^2+^-nitrilotriacetic (NTA) resin (Qiagen) pre-equilibrated with the same buffer and incubated for 1 h at 4°C under continuous agitation. The resin was washed three times using washing buffer (100 mM NaH_2_PO_4_, 100 mM Tris/HCl, and 8 M urea, pH 6.3), and rTrCβS elution was carried out using an appropriate buffer (100 mM NaH_2_PO_4_, 100 mM Tris/HCl, and 8 M urea, pH 4.5). The eluted proteins were dialysed using 50 mM NaH_2_PO_4_ pH 7.4, 300 mM NaCl and 150 mM imidazole overnight at 4°C. The purity of the recombinant protein was then assessed by SDS-PAGE, and its concentration was determined by the Bradford method (Bio-Rad) using BSA as a standard. The protein was stored at -20°C.

To obtain recombinant CS (rTrCS), different approaches were assessed. pET14-TrCS was introduced into *E. coli* BL21 (DE3), BL21 (DE3)pLysS, and Rosetta strain, and expression was induced using different IPTG concentrations (0.2, 0.5, or 1.0 mM) and temperatures (15°C, 25°C, or 37°C). Despite the number of experimental conditions tested, it was not possible to obtain recombinant TrCS.

### Production of α-rTrCβS mouse polyclonal antibodies

Approximately 50 μg of purified rTrCβS (44 kDa) was subcutaneously inoculated into Balb/C mice using Alu-Gel (Serva) as an adjuvant. Each mouse received four consecutive inoculations at 12-day intervals, with monitoring for an antibody response using enzyme-linked immunosorbent assay (ELISA) with rTrCβS as the antigen.

### Comparative analysis of CβS expression by *T. rangeli* and *T. cruzi*

Quantification of CβS expression was performed using soluble protein fractions from *T. rangeli* and *T. cruzi*. A total of 1 × 10^8^ epimastigotes or trypomastigotes were washed once with D-PBS and lysed by repeated aspiration in ice-cold lysis buffer (0.25 M sucrose, 0.25% Triton X-100, and 10 mM EDTA) containing a protease inhibitor cocktail (Sigma-Aldrich). Cellular debris was removed by centrifugation at 12,000 × *g* for 20 min at 4°C [22]. The protein concentrations in the extract were determined by the Bradford method (Bio-Rad) using BSA as a standard and stored at -20°C.

Soluble protein extracts (30 μg) of the different life cycle stages of *T. rangeli* and *T. cruzi* were fractionated on 12% SDS-PAGE and electroblotted onto nitrocellulose membranes (GE Healthcare) in an appropriate buffer (25 mM Tris; 192 mM glycine; 20% v/v methanol, pH 8.3). The membranes were then blocked with 5% non-fat milk in blotting buffer (25 mM Tris–HCl pH 7.4, 150 mM NaCl, and 0.1% Tween-20) overnight at 4°C [23]. After blocking, the membranes were incubated for 1 h with an anti-rTrCβS mouse polyclonal antiserum (1:4,000) or anti-α tubulin monoclonal antibody (1:10,000) used as a loading control. After washing, the membranes were incubated with anti-mouse IgG conjugated to horseradish peroxidase (1:10,000), followed by washing and detection on radiographic films using an ECL kit (Pierce) according to the manufacturer’s recommendations. The western blots were digitally analysed using the software package Image J 1.463r, subtracting the background of each blot prior to measuring the intensity of specific bands. Integrated densities for each band were determined for each protein of interest and its corresponding loading control. The ratio of the band intensity of the protein of interest versus the band intensity of the corresponding loading control was used as the relative protein expression level and allowed the comparison with other samples.

### Enzymatic assays for CβS and CS activities

#### *Cystathionine β-synthase*

The assay method described by Walker and Barret was used [24]. Briefly, the reaction mixture contained 70 μmol Tris–HCl buffer (pH 8.4), 0.4 mM PLP, and 1.5 μg/μL of total protein extract from parasites or 0.1 μg/μL of rTrCβS (as a positive control) in a final volume of 100 μL. In the case of the protein extract, the mixture also contained 0.1 mM CuSO_4_ to inhibit cystathionase activity. All components were equilibrated for 2 min at 37°C, and the reaction was initiated by the addition of 40 mM D,L-homocysteine and 20 mM L-serine. The reaction was stopped 45 min later by the addition of 100 μL 50% (w/v) trichloroacetic acid. The precipitated protein was removed by centrifugation at 12,000 × *g* for 5 min, and the amount of cystathionine was determined by adding 1 mL of acid-ninhydrin reagent (1 g ninhydrin dissolved in 100 mL concentrated acetic acid and 1/3 volume of phosphoric acid) to 100 μL of the assay supernatant fraction. The mixture was then boiled for 5 min, cooled for 2 min on ice, and incubated for 20 min at room temperature (25°C) for colour development. The absorbance was measured at 455 nm. Each enzymatic assay was performed including negative controls (all reagent components without enzyme or without substrate). A standard curve was prepared using 0–3 μmol of cystathionine dissolved in acid-ninhydrin reagent and treated as described above to quantify the amount of cystathionine formed [25].

#### *Cysteine synthase*

The CS activity in the total protein extracts from parasites (1.5 μg/μL) or bacteria (positive control) was determined by measuring cysteine production at 37°C in a 500 μL reaction containing 200 mM potassium phosphate buffer (pH 7.5), 10 mM DTT, 0.2 mM PLP, 6.5 mM *O-*acetylserine (OAS), and 4 mM sodium sulfide (Na_2_S). All the components except sodium sulfide were pre-incubated for 5 min at 37°C; the reaction was initiated by the addition of sodium sulfide and incubated for another 30 min and then stopped using 50 μL of 20% trichloroacetic acid (w/v). The mixture was centrifuged for 5 min at 12,000 × *g*, and the supernatant was used for cysteine analysis, as previously described with some modifications [26]. Briefly, an aliquot (500 μL) of the supernatant was added to 500 μL of ninhydrin reagent (250 mg ninhydrin dissolved in 10 mL concentrated acetic acid: concentrated HCl, 60 ~ 40 v/v). The mixture was boiled for 10 min and immediately cooled on ice before the addition of 500 μL of 95% (v/v) ethanol. The amount of cysteine formed was determined by measuring the absorbance of the reaction mixture at 560 nm [27]. Each enzymatic assay was performed including negative controls (all reagent components without enzyme or without substrate). A standard curve was prepared with L-cysteine (0–1 μmol) dissolved in ninhydrin reagent and treated as described above to quantify the amount of cysteine formed. The serine sulfhydrylase activity of CS was determined in the same way as described for the CS assay above, except that 6.5 mM serine was used instead of OAS.

### Cellular thiol contents

The total thiol content of *T. rangeli* and *T. cruzi* epimastigotes was determined using deproteinised parasite extracts prepared as formerly described [28]. Epimastigotes in the exponential phase (1 × 10^8^ parasites/mL) were harvested, washed with D-PBS, and suspended in 0.6 mL of 25% trichloroacetic acid. After 10 min on ice, the denatured proteins and cell debris were removed by centrifugation at 13,000 × *g* for 10 min at 4°C. The thiol content of the supernatant solution was determined by Ellman’s method [29] using 0.6 mM 5,5′-dithio-bis(2-nitrobenzoic acid) (DTNB) in 0.2 M sodium phosphate buffer (pH 8.0). The concentration of DTNB derivatives of thiols was estimated spectrophotometrically at 412 nm. Calibration curves were performed with known amounts of cysteine.

### Epimastigote susceptibility to oxidative and nitrosative stress *in vitro*

Parasite susceptibility to oxidative or nitrosative stress was assessed using Alamar blue (AB) assays, as described elsewhere [22,30] with minor modifications. Briefly, 5 × 10^5^ *T. rangeli* and *T. cruzi* epimastigotes were incubated for 48 h with 100 μL parasite culture in quadruplicate in 96-well plates. Aliquots of 100 μL of 30% hydrogen peroxide (Sigma-Aldrich) or S-nitroso-N-acetylpenicillamine (SNAP, Molecular Probes®- Life Technologies) prepared at different dilutions (0–100–150–300–500–1000–1500 μM and 0–5–20–50–150–300–500–1000 μM, respectively) were added, as reported [22,31]. After incubation at 26°C for 24 h, 20 μL of AB reagent (Invitrogen) was added to each well to assess parasite viability via fluorescence emission at 600 nm. Data from treated and non-treated cultures were used to calculate the IC_50_ by a sigmoidal regression analysis (with variable slope) using GraphPad Prism v.5.0. Untreated control parasites and reagent blanks were included in each test plate.

### Statistical analysis

All experiments were performed in triplicate, and the results are presented as the mean and the standard deviation (SD) or standard error of the mean (SEM). Normalised data were analysed by a one-way ANOVA followed by Bonferroni post-tests or Student’s *t*-test, as indicated in the figure legends, using the software GraphPad Prism v.5.0.

### Ethical approval

The procedures involving animals were previously approved by the UFSC Ethics Committee on Animal Use – CEUA (Reference number: 23080.025618/2009-81).

## Results

### The *T. rangeli* genome contains genes encoding CβS and CS enzymes

Using the nucleotide and protein sequences of CβS and CS orthologs from plants, bacteria, yeast, and parasitic protozoa as queries, a search of *T. rangeli* genome and transcriptome databases allowed the identification of genes encoding CβS and a partial gene sequence for CS. Additionally, the *T. rangeli* genome contains a single copy of the cystathionine γ-lyase (CGL) gene of the RTS pathway but lacks the genes encoding serine acetyltransferase (SAT) present in the *de novo* biosynthetic pathway of other trypanosomatids. The sequences for CβS and CS were then back-searched using the SWISSPROT and NCBI databases, which confirmed the identity of both genes. These results suggest that, as in other trypanosomatids, *T. rangeli* possesses genes coding for the enzymes involved in these two cysteine biosynthetic routes: CβS in the transsulfuration pathway and CS in the *de novo* biosynthesis pathway.

**Figure 1:**
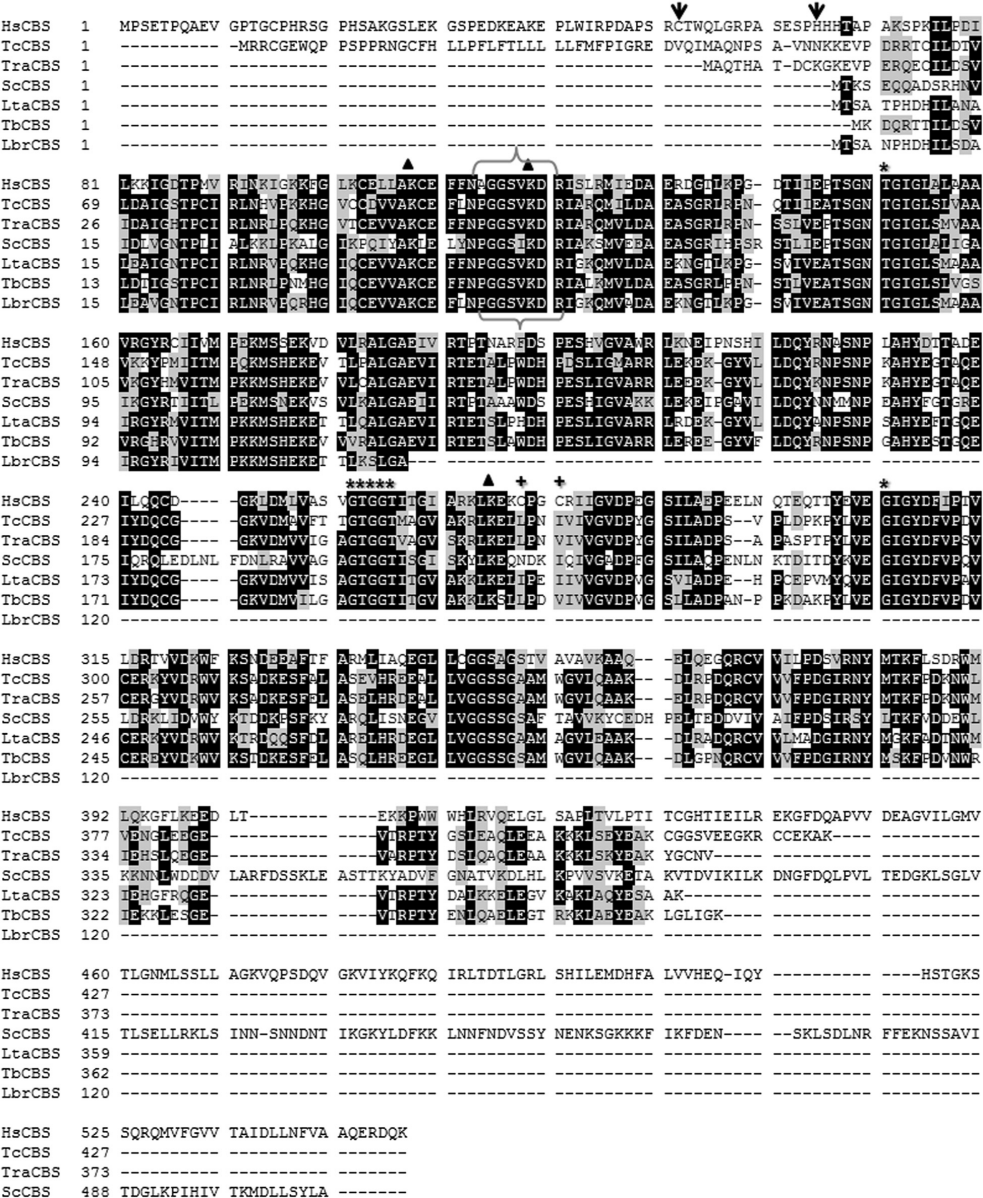
**Multiple alignment of deduced amino acid sequences of CβS from *T. rangeli *(TrCβS) and other representative organisms.** The identity (black background) and conservation (grey background) of the amino acid residues are shown. Brackets indicate the consensus amino acid residues of the putative pyridoxal phosphate-binding motif (PXXSVKDR), and other motifs vital for CβS activity are indicated with asterisks (*). The oxido-reductase motif of HsCβS is highlighted with (**+**). The lysine residues required for CS catalytic activity are marked with triangles. The positions of the heme-binding residues within the heme domain of the human CβS enzyme (Cys^52^ and His^65^) are marked with (**↓**). HsCβS: Human (P35520); TcCβS: *Trypanosoma cruzi* (Tc00.1047053511691.20); ScCβS: *Saccharomyces cerevisiae* (P32582) LtaCβS: *Leishmania tarentolae* (LtaP17.0270); TbCβS: *Trypanosoma brucei* (Tb11.02.5400); LbrCβS: *Leishmania braziliensis* (LbrM.17.0230).

After cloning and sequencing, it was found that *T. rangeli* CβS (*TrCβS*) predicts a protein of 373 amino acids (44 kDa) that reveals high sequence identity with CβS from *T. cruzi* (84%), *T. brucei* (78%), and *L. major* (75%) compared to human CβS (50%). Multiple sequence alignment confirmed that TrCβS contains three out of the four lysine residues (Lys ^53^, Lys^64^, Lys^213^) required for CS activity; the consensus sequence for the putative cofactor pyridoxal phosphate-binding domain is highly conserved. rTrCβS, as well as CβS from other trypanosomatids, differs from *H. sapiens* CβS (HsCβS) by lacking the haem-binding (redox sensor) and oxidoreductase motifs (Cys XX Cys) at the N- and C-termini, respectively (Figure 1).

**Figure 2:**
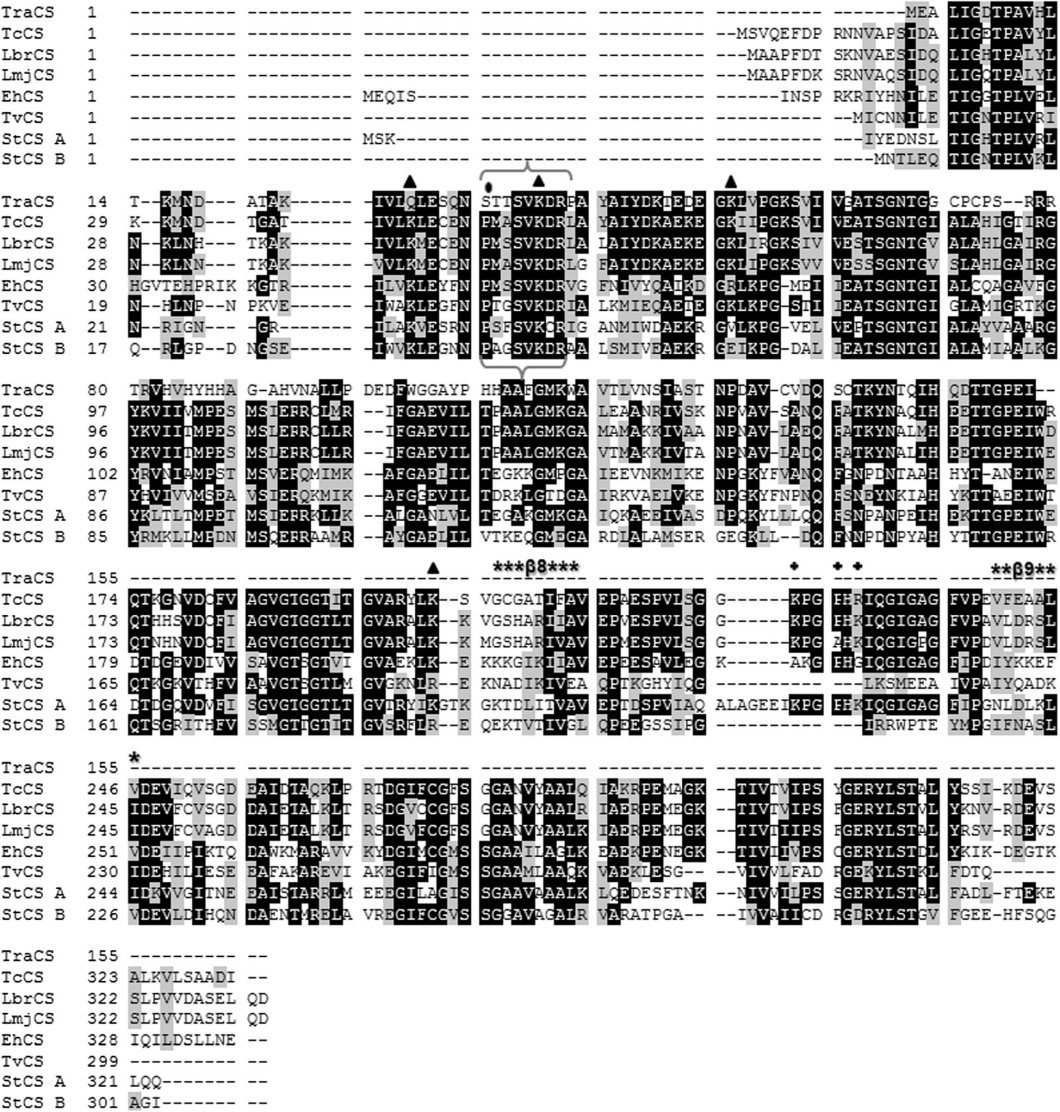
**Multiple alignment of deduced amino acid sequences of CS from *T. rangeli *and other representative organisms.** The identity (black background) and conservation (grey background) of the amino acid residues are shown. Brackets indicate the consensus amino acid residues of the putative pyridoxal phosphate-binding motif (PXXSVKDR); the substitute for the proline residue is marked with (•), and the lysine residues required for cysteine synthase activity are indicated with triangles. The β8–β9 loop at the entrance of the active site is indicated with an asterisk (*), and the positively charged residues involved in binding with SAT are indicated with (**+**). TraCS: *Trypanosoma rangeli*; TcCS: *Trypanosoma cruzi* (Tc00.1047053507165.50); LbrCS: *Leishmania braziliensis* (LbrM.35.3820); LmjCS: *Leishmania major* (LmjF.36.3590)*;* EhCS: *Entamoeba histolytica*; TvCS: *Trichomonas vaginalis* (XP001325874); StCS A: *Salmonella typhimurium* CysK (P0A1E4); StCS B: *Salmonella typhimurium* CysM (NP_456975).

The *T. rangeli* CS gene (TrCS) encodes a protein of 155 amino acids (~16.8 kDa) that is 53% identical to the *T. cruzi* ortholog but exhibits lower identity with *L. major* (46%) and *L. infantum* (45%). Although CβS and CS are evolutionarily related enzymes, we found a low identity between TrCβS and TrCS (≤13%) when compared to the TrCS identity with the other orthologues from plants and bacteria (~31-33%). An analysis of the predicted amino acid sequences of TrCS revealed an amino acid change of Pro^32^ → Ser within the putative pyridoxal phosphate-binding domain (PXXSVKDR). Unlike other CSs, TrCS has only two of the four lysine residues (Lys^37^, Lys ^53^) shown to be important for the catalytic activity of the enzyme. Furthermore, TrCS does not have the canonical β8-β9 loop described in CS enzymes, which is important for access to the active site, and neither of the positively charged residues (Lys-His-Lys) involved in binding with serine acetyl-transferase (SAT) (Figure 2).

### Stage-specific expression of CβS in *T. rangeli*

**Figure 3:**
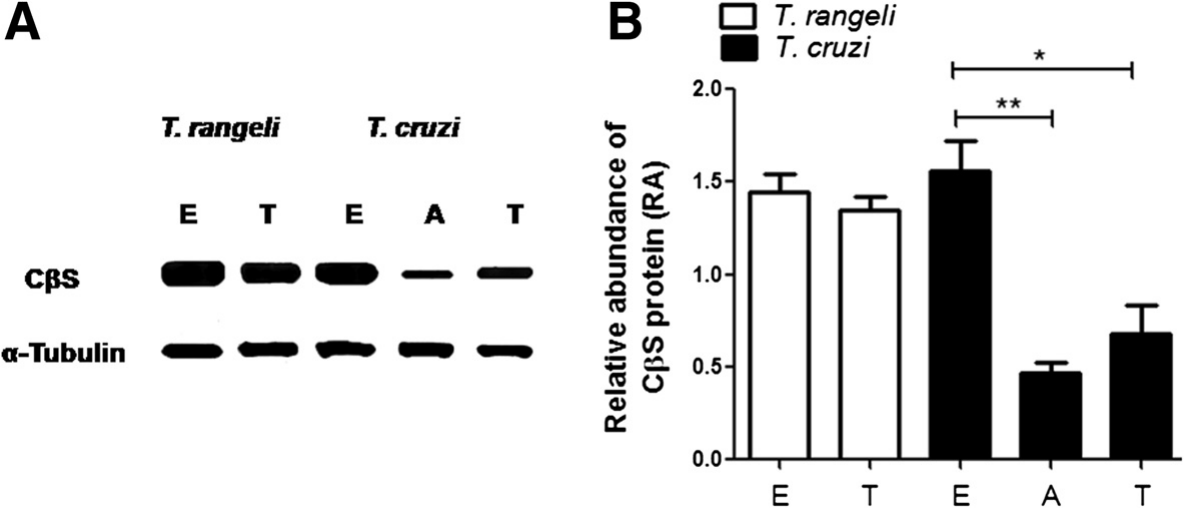
**Protein expression levels of CβS in *T. rangeli *and *T. cruzi*. A**. Western blot analysis of soluble extracts obtained from epimastigotes (E), and trypomastigotes (T) of *T. rangeli* and *T. cruzi*, and amastigotes (A) of *T. cruzi*. **B**. Densitometric analysis of CβS expression using ImageJ software and significant differences in CβS expression between epimastigotes and trypomastigotes, as determined by the t-test (*P < 0.05, **P < 0.01). The normalisation of protein loading was performed by the immunodetection of α-tubulin.

The relative abundance of the CβS protein was evaluated in *T. rangeli* epimastigote and trypomastigote forms by western blotting, showing no significant differences between the forms. The absence of TrCβS stage-specific expression contrasts with the homologous protein in *T. cruzi* (TcCβS), for which the expression level of CβS was found to be significantly increased in epimastigotes (Figure 3A, B).

### CβS is active in *T. rangeli*

**Figure 4:**
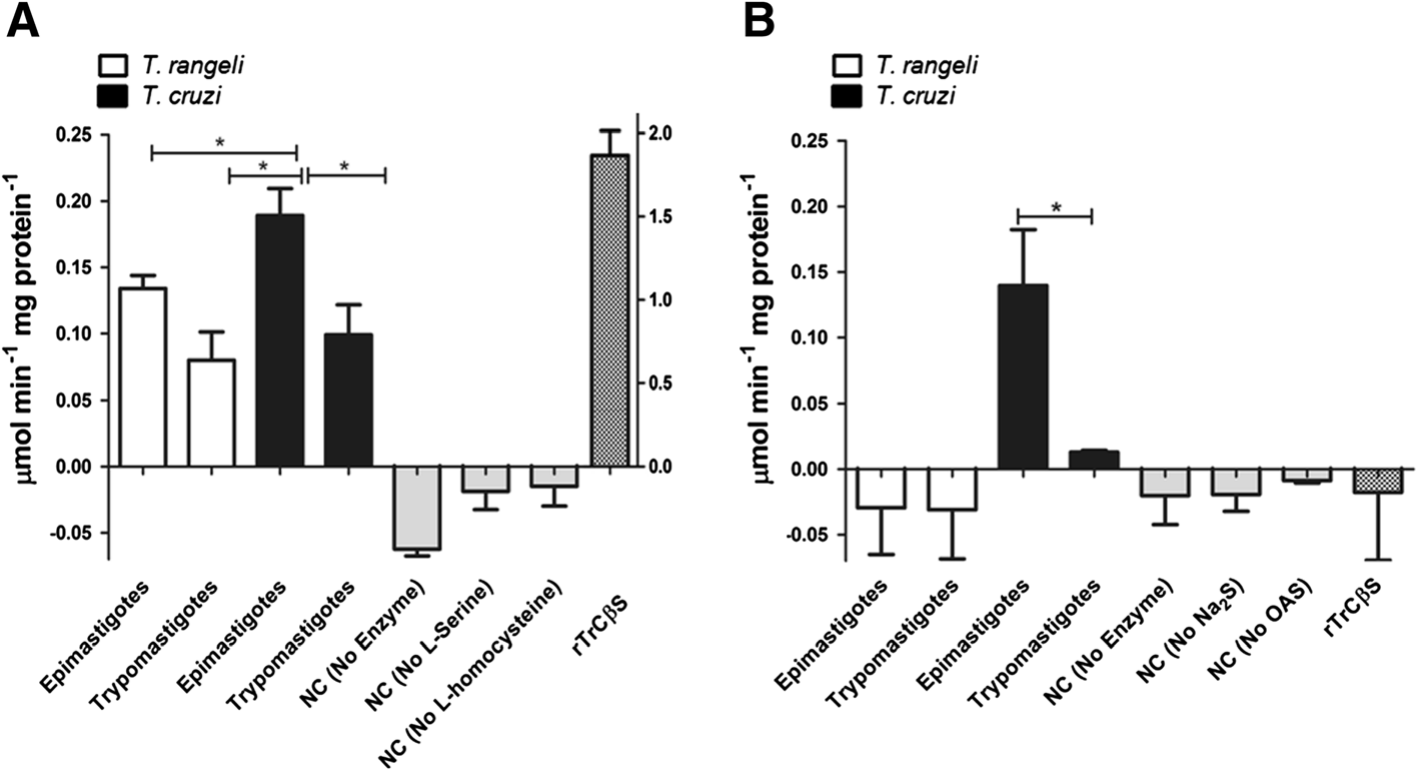
**Detection of CβS and CS activities in protein extracts of *T. rangeli *and *T. cruzi *epimastigotes and trypomastigotes. A**. The activities of CβS were determined in soluble extracts from trypanosomes using the recombinant enzyme rTrCβS as a positive control (axis Z). The results represent the average of five independent experiments performed in triplicate ± SD. **B**. The activities of CS were determined in soluble extracts from trypanosomes. The data represent the mean of five independent experiments performed in triplicate ± SD. Significant difference (*P < 0.05). NC = negative controls.

The enzymatic studies on *T. rangeli* extracts showed that CβS activity is detectable in both epimastigotes (0.13 μmol min^-1^ mg^-1^) and trypomastigotes (0.079 μmol min^-1^ mg^-1^ of protein) (Figure 4A), whereas CβS activity was 1.9 times higher in the extracts from *T. cruzi* epimastigotes versus trypomastigotes. Conversely, CS activity was undetectable in the protein extracts from both *T. rangeli* forms (Figure 4B).

rTrCβS showed CβS activity of 2.2 ± 0.2 μmol min^-1^ mg^-1^ of protein (Figure 4A), with a *km* of 1.702 ± 0.11 mM for L-serine and a *Km* of 7.301 ± 1.9 mM for L-homocysteine, indicating a high binding affinity for L-serine and a weak binding affinity for L-homocysteine. rTrCβS was also capable of generating L-cysteine from serine and sodium sulfide, but with a very low specific activity (serine sulfhydrylase activity of 0.013 μmol min^-1^ mg^-1^ of protein). Different from *T. cruzi* CβS, rTrCβS did not show any CS activity (data not shown).

### Total thiol content in *T. rangeli* and *in vitro* oxidative/nitrosative stress phenotyping

**Figure 5:**
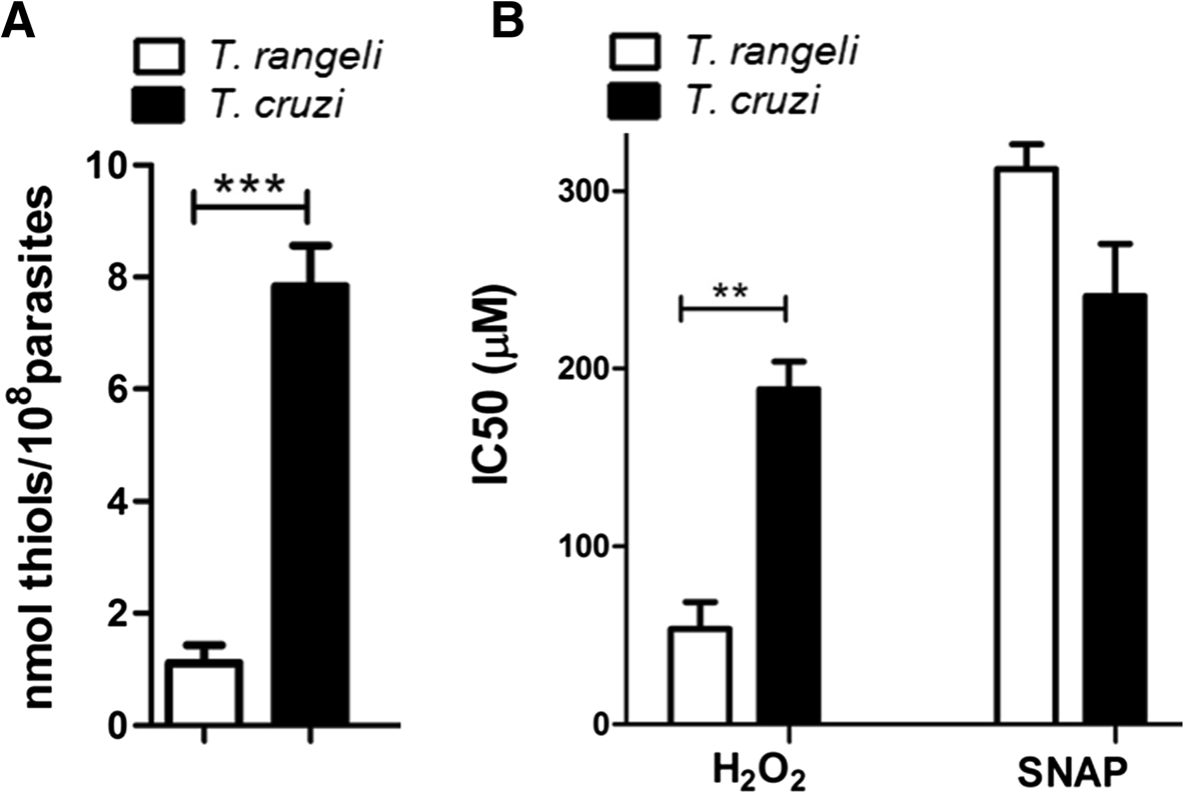
**Total thiol content and effects of oxidative and nitrosative stress on *T. rangeli *and *T. cruzi *viability. A**. The total thiol content was determined in soluble extracts obtained from the epimastigote form. Error bars represent the SEM of three independent experiments. **B**. *In vitro* susceptibility of epimastigotes of *T. rangeli* and *T. cruzi* exposed to oxidative stress by hydrogen peroxide (H_2_0_2_) or nitrosative stress by S-nitroso-N-acetylpenicillamine (SNAP). Error bars represent the SEM of three independent experiments, performed in quadruplicate. Significant differences were detected by a one-way ANOVA, followed by Bonferroni post-tests (**P < 0.01, ***P < 0.001).

A comparative analysis of the total thiol levels of *T. rangeli* and *T. cruzi* revealed significant differences between these parasites. *T. cruzi* showed a thiol content of 7.8 nmoles/10^8^ parasites, whereas *T. rangeli* had a thiol content that was almost seven times less (1.1 nmoles/10^8^ parasites) (Figure 5A).

Based on these results, the *T. rangeli* susceptibility to oxidative and nitrosative stress was evaluated by subjecting epimastigotes to stress conditions *in vitro* with H_2_O_2_ or SNAP. This parasite was found to be more sensitive than *T. cruzi* to oxidative stress (H_2_O_2_), showing an IC_50_ of 53 μM, which is significantly less (P < 0.01) than the IC_50_ obtained for *T. cruzi* epimastigotes (188.3 μM). Nevertheless, the difference between these parasites was less pronounced under nitrosative stress conditions (SNAP), with *T. rangeli* being more resistant than *T. cruzi* (IC_50_: 312 μM and 240.7 μM, respectively) (Figure 5B).

## Discussion

Our results indicate that RTS appears to be the only pathway for cysteine biosynthesis in *T. rangeli*. At the genomic level, *T. rangeli* contains single copies of genes coding for the CβS and CGL (cystathionine γ-lyase) enzymes of the RTS pathway but lacks genes encoding a protein of the cysteine *de novo* biosynthetic pathway (SAT). Additionally, a partial gene sequence for CS was found that has an A-G nucleotide transition at position 470, which generates a stop codon (TAG) (data not shown); thus, the truncated protein encoded lacks two of the four lysine residues required for CS activity.

A biochemical analysis of rTrCβS showed a higher CβS activity compared to hsCβS for generating cystathionine via the condensation of L-serine and L-homocysteine, though rTrCβS is less active than TcCβS [32-34]. In spite of this, the binding substrate affinity was comparable to the affinity of the CβS enzyme from *L. major* and humans [9]. Similar to other CβSs, rTrCβS can also form cysteine from L-serine and sodium sulfide, but is unable to utilise OAS and sulfide to catalyse the production of cysteine. Nevertheless, inter-species variations in other CβS catalysed reactions [24] could explain the absence of CS activity mediated by TrCβS.

The presence of a truncated CS gene as revealed by high-quality sequencing (Phred ≥50), and the absence of CS activity in both epimastigote and trypomastigote extracts suggests that the *de novo* cysteine biosynthetic pathway is absent or not functional in *T. rangeli*. Nevertheless, *T. rangeli* possesses a functional RTS pathway, a characteristic shared with *T. brucei,* for which only CβS activity has been reported in bloodstream trypomastigote extracts but at a very low level [35]. This result indicates that similarities in the metabolism of sulfur-containing amino acids exist between *T. rangeli* and *T. brucei,* another parasite that does not possess an intracellular mammalian host stage. Such findings may suggest that the extracellular stage of the life cycle of parasitic protozoa and the RTS biosynthetic pathway are causally connected.

No stage-specific association was found for *T. rangeli* CβS activity and protein levels, contrasting with *T. cruzi*, with epimastigotes (insect-form) that present significantly higher activity and protein levels. Other studies on the RTS pathway in *T. cruzi* have demonstrated the same stage-specific regulation of this pathway and have shown a likely association with the complex life cycle of this parasite and the availability of sulfur-containing amino acids in different parasite environments [33,34].

We found significantly lower levels of total thiol content in *T. rangeli* compared to *T. cruzi* epimastigotes. Based on the fact that cysteine forms the basic building block of all thiol antioxidants [2], one possible explanation for the lowest thiol levels observed may be because *T. rangeli* only uses the RTS pathway as a cysteine biosynthesis source. Another important aspect is related to the fact that exogenous organic sulfur-containing amino acids can be supplied by transporters [3,9,36]. However, such a mechanism and its possible influence on the total thiol levels in *T. rangeli* remain to be explored.

Different from *T. cruzi*, which faces oxidative stress in the mammalian host and within the triatomine vector’s digestive tract, *T. rangeli* is exposed to further oxidative and nitrosative stress while reaching the triatomine hemolymph and salivary glands [37]. Recently, studies have demonstrated the activation of the vector immune system during *T. rangeli-Rhodnius prolixus* interactions, including the generation of nitric oxide and superoxide free radicals [38-40]. The greater resistance of *T. rangeli* to SNAP compared to *T. cruzi* could be explained by the ability of *T. rangeli* to modulate insect immune/cellular factors [38,41], especially those related to nitrosative production, thus allowing the parasite to survive and multiply freely in the insect’s hemolymph and to invade and complete its development within the salivary glands [42].

Because thiols have been demonstrated to be the central metabolites in the redox metabolism of several parasite species [43], thus playing an important role in protection against oxidative stress, the higher *T. rangeli* susceptibility to hydrogen peroxide may be due its reduced total thiol content. In addition, the absence of an active CS enzyme potentiates the *T. rangeli* susceptibility to hydrogen peroxide, leading to the death of the parasite. Such findings are in agreement with reports in amoebae, whereby the overexpression of CS increases the total cellular thiol content and the resistance to oxidative stress due to hydrogen peroxide [8].

## Conclusion

These findings demonstrate that the RTS pathway is active in *T. rangeli*, suggesting that this may be the only pathway for cysteine biosynthesis in this parasite because no CS activity was detected in epimastigotes and trypomastigotes and the CS genes are truncated due to the presence of stop codons. In this sense, the RTS pathway would have an important functional role during the insect stage of the life cycle of this protozoan parasite.

## Competing interests

The authors declare that they have no competing interests.

## Authors’ contributions

IR and JT participated in the conception and design of the study and wrote the manuscript. LY was involved in cloning CβS. MS, AR, and EG were involved in the conception of the study and wrote the manuscript. All authors read and approved the final version of the manuscript.
